# Cathelicidin regulates goblet cell mucus secretion and mucus-associated proteins in *Citrobacter rodentium*-induced colitis

**DOI:** 10.1080/19490976.2025.2538696

**Published:** 2025-07-30

**Authors:** Niloofar Mirzadzare, Graham A. D. Blyth, Rita Hannawayya, Karina M. Cirone, Geeta Kilari, Priyoshi Lahiri, Yi Lin Tan, Aydin Ivan Herik, Yongpeng Fu, Hayley Gorman, Björn Petri, Kris Chadee, Eduardo R. Cobo

**Affiliations:** a Faculty of Veterinary Medicine, University of Calgary, Calgary, Alberta, Canada; b Microbiology, Immunology and Infectious Diseases, Cumming School of Medicine, University of Calgary, Calgary, Canada; c The Calvin, Phoebe and Joan Snyder Institute for Chronic Diseases, University of Calgary, Calgary, Canada

**Keywords:** Colonic epithelium, cathelicidin, goblet cells, mucus, *citrobacter rodentium*, reactive oxygen species

## Abstract

Colonic goblet cells play a crucial role in mucosal defense by secreting Muc2 mucin and other proteins that entrap and expel enteropathogens. However, the role of innate effectors in the gut like cathelicidin peptides in regulating the mucus barrier during infections remains unclear. In this study, we used cathelicidin-deficient (*Camp*^*-/-*^) littermates, colonoids, and human LS174T goblet-like cells to investigate how cathelicidin modulates goblet cell function and mucosal defense against attaching/effacing enteropathogen *Citrobacter rodentium*. We showed that increased fecal shedding and epithelial colonization by *C. rodentium* in *Camp*^*-/-*^ littermates was accompanied by impaired mucus secretion and higher retention of mucin granules and trefoil factor 3 (Tff3) in bloated colonic goblet cells. Reduction in mucus secretion by goblet cells was accompanied by reduced reactive oxygen species (ROS) production during *C. rodentium* infection in *Camp*^*-/-*^ as compared to *Camp*^*+/+*^ littermate controls. In LS174T goblet-like cells, human cathelicidin LL-37 stimulated the secretion of TFF3 and resistin-like molecule β (RELMβ) in a ROS-dependent manner. These findings reveal that cathelicidin regulates goblet cell mucus and mucus-associated protein secretion through a ROS-mediated mechanism critical for bacterial clearance and maintenance of gut homeostasis.

## Introduction

The secretion of mucus glycoproteins, preformed and stored in granules by colonic goblet cells, is a central innate defense mechanism during acute colitis caused by attaching/effacing (A/E) enteropathogenic bacteria. Colonic mucus comprises a dense inner layer impenetrable to bacteria and a loosely attached outer layer colonized by indigenous microbiota.^1^ Mucus protects the gut by lubricating the lumen, preventing mechanical damage, and spatially separating the microbiota from the epithelium while expelling pathogenic microbes.^1^ Enteropathogenic A/E bacteria can subvert the mucus barrier and intimately attach to the colonic epithelium, inducing colitis.^2^ A leading example is the murine-specific A/E pathogen *C. rodentium*, which penetrates the mucus layer by expressing “lytic” virulent factors, including a protein involved in intestinal colonization (Pic) and a serine protease autotransporter of *Enterobacteriaceae* (SPATE) with mucinase activity.^3^ During colonization, *C. rodentium* adheres to the apical plasma membrane of goblet cells, forming A/E lesions and depleting mucus cargo.^4^ *Muc2*^*-/-*^ mice are highly susceptible to *C. rodentium* infection and gut pathology.^2,5^ Mucolytic commensal microbiota and metabolites produced by certain bacteria, e.g., *Bacteroides thetaiotaomicron*,^6^ may further facilitate *C. rodentium* colonization.^7^

Colonic mucus consists of glycoprotein MUC2 mucin and various goblet cell-associated proteins secreted apically into the intestinal lumen. Goblet cells produce trefoil factor (TFF)3,^8^ a small protease-resistance peptide within the theca that is secreted independently of MUC2 mucin.^9^ TFF3 aids epithelial restitution and mucosal healing^10,11^ by mechanisms that include epithelial autophagy,^12^ migration during healing processes,^13^ and prevention of apoptosis.^14^ The production of TFF3 is reduced during *C. rodentium* infection.^4^ Goblet cells also release a resistin-like molecule (RELMβ), a cysteine-rich bactericidal protein encoded by the *RETNLβ* gene and regulated by commensal bacteria.^15^ RELMβ contributes to inflammation by inducing pro-inflammatory macrophages in dextran-sodium sulfate colitis (DSS)^15,16^ and CD4^+^ T cells and epithelial cell proliferation in *C. rodentium* colitis.^17^ Gene ontology (GO) enrichment analysis reveals subpopulations of goblet cells along the colonic epithelial surface that perform specific functions. “Canonical” goblet cells, expressing *Clca1* and *Fcgbp*, secrete mucus.^18^ Non-canonical cells, enriched in genes associated with lipid and amino acid metabolism, detoxification, and intestinal absorption,^18^ can act as sentinel goblet cells at the crypt entrance (neck). These goblet cells detect pathogen-associated molecular patterns (PAMPs), including Gram-negative virulent factor lipopolysaccharide (LPS), and induce downstream Muc2 secretion via Toll-like receptor (TLR) 4/MyD88, along with the synthesis of ROS.^19^ This ROS effector predisposes mucus secretion in goblet cells.^20,21^ Non-canonical intercept goblet cells express genes associated with cellular responses to stress and bacteria, likely related to a luminal-facing location.^18^

The colonic epithelium secretes into the lumen cationic peptides, including cathelicidin^22,23^ and others, such as β defensins.^24^ These peptides are supposed to accumulate under or within the sterile inner mucus layer as an antimicrobial shield^25^; however, the consequence of this interaction is unknown. Both humans and mice produce a single cathelicidin peptide. Humans produce leucine-leucine 37 amino acid residues (LL-37) encoded by the *cathelicidin antimicrobial peptide* (*CAMP*) gene. Mice produce the cathelicidin-related antimicrobial peptide (CRAMP) encoded by the *Camp* gene.^26^ Cathelicidin is secreted as a pro-peptide and cleaved by serine proteases, and the released C-terminal fragment is antimicrobial against Gram-negative bacteria.^27,28^ Cathelicidin is protective against A/E pathogens, as mice genetically deficient in cathelicidin (*Camp*^*-/-*^) exhibit exacerbated colitis and delayed clearance of *C. rodentium*^23,29,30^ and *Escherichia coli* O175:H7 ^31^. However, further research is needed to explain the diverse protective effects of cathelicidin restricted to a single anatomical niche. For instance, LL-37 is weakly microbicidal under physiological concentrations of salt, serum proteins, and lipoproteins.^31–34^ In addition, *E. coli* and *C. rodentium* have outer membrane proteases that can inactivate LL-37^35^ and CRAMP.^36^ The ability of cathelicidin to interact with cell surface receptors and intracellular proteins on neutrophils^37,38^ and non-immune cells (e.g., epithelia)^39,40^ implies cathelicidin biology involves other fine-tune innate and inflammatory roles in the gut.^41^ In the colonic epithelium, cathelicidin facilitates the internalization of *C. rodentium*/LPS and activation of endosomal TLR4, stimulating the synthesis/release of chemokine CXCL8/1^23^ and preventing epithelial apoptosis by sustaining toll-interacting protein (Tollip).^29^ It is unknown whether cathelicidin affects the mucus barrier. However, *Camp*^*-/-*^ littermates exhibit a thin colonic mucus layer,^42^ and exogenous cathelicidin increases *MUC2* gene expression in intestinal epithelial cells.^43,44^ We have previously shown that MUC2 sustains the synthesis of intestinal epithelial *CAMP* in response to the colonic parasite *Entamoeba histolytica*.^45^ Thus, goblet cells and cathelicidin seem to have potential reciprocal interactions that may have been overlooked in gut innate immunity. In this study, we identified a new role for cathelicidin in regulating goblet cell function and mucosal barrier integrity during *C. rodentium* infection through the production of ROS.

## Materials and methods

### Mice and *C.rodentium* infection

Experiments with C57/BL6 and *Camp*^*-/-*^ (B6.129X1-*Camp*^*tm1Rlg*^/J) littermates were conducted at the University of Calgary, approved by the Canadian Guidelines for Animal Welfare (CGAW) and the University of Calgary Animal Care Committee (AC20–0050), and reported according to ARRIVE guidelines https://arriveguidelines.org/arrive-guidelines. *Camp*^*-/-*^ *VillinCre* mice were genetically modified mice that express Cre recombinase under the control of the Villin 1 (Vil1) promoter. We used *Camp*^*fl/fl*^ mice, which possess loxP sites flaking the *Camp* gene (gifted by Dr. R. Gallo, UC San Diego). *Camp*^*fl/fl*^ mice were crossed to *Villin-Cre* mice (B6.Cg6. Cg-Tg(Tg (Vil1-cre)997Gum/J), which express Cre recombinase in villus and crypt epithelial cells of the small and large intestines under the control of the Villin promoter (#004586 Jackson Laboratory) to generate *VillinCre-Camp*^*fl/fl*^ mice. All mice were bred on a C57BL/6 background and kept in individually ventilated cages. Studies were conducted in males because sex differences were not observed in *C. rodentium* clearance or the severity of infection-induced colitis in mice.^46^

For infection, *C. rodentium* DBS100 or the bioluminescent *C. rodentium* ICC180 were cultured on MacConkey agar plates at 37^∘^C. Single colonies were isolated, grown overnight in Luria-Bertani (LB) broth, and cultured for 16–24 h. Mice were orally administered either PBS or *C. rodentium* (5 × 10^8^ colony-forming units, CFU) in a volume of 0.1 mL. At the indicated time points, the mice were humanely euthanized for necropsy. Bacterial shedding was assessed by quantifying *C. rodentium* (CFU/g feces) in fresh fecal pellets collected in pre-weighed tubes, homogenized in sterile PBS (0.1 mg/mL), and 10-fold serial dilutions plated on MacConkey agar plates. Our preliminary studies showed no sex difference in bacterial *C. rodentium* shedding between males and females; therefore, only males were included in the studies.

The replication of *C. rodentium in vivo* was determined using whole-body bioluminescence imaging. Mice were anesthetized with 5% (3% maintenance) isoflurane carried in 2 L O^2^/min, kept constant at 37^∘^C via air circulation, and imaged on their ventral side using an In-Vivo Xtreme 4MP imaging platform (Bruker, Billerica). Images were taken in three steps: reflectance imaging (2 s exposure time), bioluminescent imaging (60 s exposure time), and an additional X-ray imaging step (10 s exposure time). The binning was maintained at 4 × 4. Images from the In-Vivo Xtreme were acquired and analyzed using the Bruker molecular imaging software MI SE (v7.1.3.20550, Bruker, Billerica). *Citrobacter*-associated bioluminescence expression in the abdomen of *Camp*^*+/+*^ and *Camp*^*-/-*^ littermates was quantified by measuring the mean bioluminescence (after background subtraction) in a constant region of interest (ROI), which was kept constant over the imaging period. The same criteria were applied when imaging and quantifying the distal and proximal parts of the colon and the cecum *ex vivo*.

### Transmission electron microscopy of attaching and effacing lesions of colonic epithelial cells

Colonic epithelial cells and goblet cells were assessed using transmission electron microscopy (TEM). Distal colon samples were collected and fixed in 2.5% glutaraldehyde (pH 7.4) in 0.1 M sodium cacodylate buffer. The colon samples were post-fixed in 2% osmium tetroxide, dehydrated with graded acetone, and embedded in EPON resin. Sections were cut to 70 nm using a Diatome diamond knife with a UC-6 ultramicrotome (Leica), placed on 200 variable thickness mesh copper grids, baked at 60°C, and stained with 1.5% uranyl acetate and 3% lead citrate. Samples were imaged on a Hitachi H-7650 TEM at 60 kV.

### Colitis microscopical assessment

Distal colon sections (1 cm) were collected and fixed in 10% neutral buffered formalin (overnight, 4^∘^C). The tissues were dehydrated, embedded in paraffin blocks, sectioned (5 µm), and stained with hematoxylin and eosin using standard procedures. Histopathological morphological changes were blindly scored using an adapted 9-point scale^47^ that includes “%” of the cross-section affected (0 = none, 1 = 0–25%, 2 = 25–50%, 3= > 50%), changes to the epithelium (0 = none, 1 = mild crypt hyperplasia and/or mild goblet cell depletion, 2 = moderate crypt hyperplasia and/or moderate goblet cell depletion, 3 = severe hyperplasia and/or severe goblet cell depletion), and leukocyte infiltration in the lamina propria (0 = none, 1 = mild, 2 = moderate, 3 = severe).

### Goblet cell quantification in colons

Distal colon samples were fixed in Carnoy’s solution and dehydrated in ascending ethanol concentrations. Tissues were sectioned (5 μm), rehydrated, and stained with 1% Alcian blue in 3% acetic acid (Millipore Sigma) (30 min) followed by 0.5% periodic acid (10 min) and Schiff reagent (30 min). In some cases, sections were stained with 1% Alcian blue in 3% acetic acid (Millipore Sigma) for 30 min and washed extensively with distilled water before counterstaining with hematoxylin and mounting. Total goblet cells were identified and quantified blindly by two independent observers counting the number of cells per upper half of individual crypts.^48^ For each mouse, a minimum of 5–7 crypts were analyzed, and the average number of goblet cells per mouse was reported. This process was repeated for a total of 3–5 mice. Outliers were removed following the Rout test with 1%Q.

The ultrastructure of goblet cells was assessed by transmission electron microscopy (TEM). Distal colon samples (1 cm) were collected and fixed in 2.5% glutaraldehyde buffered to pH 7.4 in 0.1 M sodium cacodylate buffer. The tissues were post-fixed in 2% osmium tetroxide, dehydrated with graded acetone, infiltrated with several graded changes of EPON exposure resin: acetone, and embedded in EPON resin. Sections were cut at a thickness of 70 nm on a Diatome diamond knife using a UC-6 Ultramicrotome (Leica). Sections were picked up on 200 variable thickness mesh copper grids, baked at 60°C, and stained with 1.5% uranyl acetate and 3% lead citrate before imaging on a Hitachi H-7650 TEM at 60 kV.

### Intestinal microbiome fluorescence in situ hybridization (FISH) analysis

Colonic tissues containing fecal pellets were collected, fixed in cold Carnoy’s fixative (4 h), transferred to 100% ethanol before paraffin embedding, and sectioned (5 µm). After deparaffinization and rehydration, FISH was performed by hybridization with a Cy5-EUB338-I probe (5’-GCTGCCTCCCGTAGGAGT-3’) (2.5 ng/mL, overnight, 37°C). Sections were stained with DAPI before mounting and imaging using an I×71Olympus fluorescence microscope.

### Cell culture and cathelicidin stimulation

Human adenocarcinoma colonic-like goblet cells (LS174T) were cultured in Eagle’s Minimum Essential Medium (EMEM) supplemented with 10% fetal bovine serum (FBS), 20 mM *N*-2-hydroxyethylpiperazine-N-2-ethane sulfonic acid (HEPES), and penicillin/streptomycin (100 U/mL). Murine bone marrow-derived macrophages were isolated at 6–8 weeks old C57BL/6 male mice. Healthy mice were euthanized, and bone marrow was collected from the femurs and centrifuged at 300 × *g* for 5 min. The bone marrow pellet was suspended in BMM medium, composed of RPMI 1640 medium supplemented with FBS (10%), penicillin and streptomycin (1%), β-2-mercaptoethanol (0.1%, 55 mM), and mouse fibroblast (L-929)-conditioned media (10%). L-929-conditioned media were used as a source of macrophage-colony stimulating factor (M-CSF) seeded in T75 culture flasks with 50 mL of RPMI 1640 supplemented with FBS (10%) (Gibco), penicillin and streptomycin (1%) (Gibco), and β-2-mercaptoethanol (0.1%, 55 mM) (Gibco). These cells were incubated for 7 days at 37°C in a 5% CO2 atmosphere, and the supernatant containing M-CSF was collected and filtered sequentially through 45 μm (97066–206, VWR) and 20 μm filters (97066–200, VWR). Bone marrow was seeded in 96-well plates with BMM for 6 days for differentiation. Cells seeded (1x 10^5^ cells in 24-well plates) and cultured until 90% confluence were washed once with PBS and stimulated with LL-37 (Bachem; 4046855.0005), scLL-37 (Bachem; 4099707.1000), or CRAMP (Bachem; 4056438) in serum-free EMEM at the indicated concentrations and times. RNA and supernatants were collected for gene and protein analysis.

### Murine colonoids

Murine colon spheroids were derived from *Camp*^*+/+*^ and *Camp*^*-/-*^ littermates. Colons were taken from 8-week-old mice, opened longitudinally, and washed with PBS. Colonic crypts were collected by gentle dissociation using a dissociation buffer (StemCell Technologies), and approximately 100 crypts were embedded in Matrigel (Corning). Colonoids were cultured in a 50% conditioned medium containing EGF (50 ng/mL), N-acetylcysteine (1 mM), and primocin (10 µg/mL). Colonoids were fed every other day for 7 days and cultured in the presence of DAPT (5 µM) for 1 day to drive goblet cell differentiation. Colonoids were washed in serum-free media and fixed in 4% PFA (30 min) to dissolve the Matrigel.

### Lectin immunohistochemistry for mucin glycans

To image the spatial distribution of mucin glycans in distal colons and colonoids, samples fixed in Carnoy’s solution were sectioned (5 μm) and subjected to a thorough deparaffinization process. Samples were washed twice in Neo-Clear (5 min) followed by a series of ethanol washes (100% ethanol twice, then 95%, 70%, and 50% ethanol, each for 3 min) and washes in distilled water (5 min). Slides were permeabilized (10 min with 0.2% Triton-X100) and blocked in Tris-buffered saline (TBS) containing 2% bovine serum albumin (BSA). For primary blotting, samples were incubated with Ulex europaeus agglutinin I conjugated to fluorescein isothiocyanate (UEA I-FITC, Sigma Aldrich) (1:500), Wheat Germ Agglutinin conjugated to Alexa647 (WGA-Alexa647, Sigma Aldrich) (1:500), and DAPI (1:1,000) nuclear markers (RT, 30 min with gentle agitation). Each treatment group included a control slide stained only with DAPI. Slides were washed and mounted with coverslips sealed with ProLong™ Gold antifade mounting solution (ThermoFisher). Slides were imaged and video-recorded using a Nikon A1R confocal microscope (60X). UEA-I^+^ and WGA^+^ signals were quantified by measuring the percent (%) positive area of staining on Image J. A minimum of 5 images per mouse were scored, and the average scores were reported for 3–5 mice per treatment group.

### Flow cytometry analysis of immune cells in the colon

Distal colon samples were collected from PBS and C. rodentium-challenged mice at the peak of infection. Lamnia propria cells were isolated using a dissociation kit (130–097–410 Miltenyi Biotech) and stained with eBioscience™ Fixable Viability Dye eFluor™ 780, Spark Blue™ 550 anti-mouse CD45, BD Horizon™ BUV563 anti-mouse Ly-6 G, BD Horizon™ BV711 anti-CD11b, BD OptiBuild™ BV605 anti-mouse Siglec-F, and BD OptiBuild™ BUV661 anti-mouse F4/80 antibodies. Cells were fixed using Thermofisher Foxp3 Fixation/Permeabilization, run on a Cytek Aurora cytometer, and analyzed with FlowJo.

### Transcriptional gene expression of innate effectors in the colon

Gene expression of innate effectors and cytokines was quantified by quantitative real-time polymerase chain reaction (qPCR) using primers for human *MUC2*, *RETNLB*, *TFF3* and murine *Muc2*, *mReg3γ*, *Tnfα*, *Il1β*, *Ifnγ*, *Relmb*, *Tff3*, *Il17, Cldn2*, and *mIl22* Table 1. Total RNA was isolated using conventional methods (Ribozol^TM^ RNA extraction), and total RNA (1 µg) was used to synthesize cDNA (qScript cDNA synthesis kit, Quantabio). Target gene mRNA values were corrected relative to housekeeping human *HPRT* and murine *Hprt* genes, and efficiency ( > 95%) ensured the amplification of a single product of the correct size, as indicated in the MIQE guidelines.^49^ Data were analyzed using the 2^−ΔΔCT^ method and reported as mean fold change of target transcript levels in stimulated groups versus the untreated control group.

**Table 1.** Primers used for qPCR analysis.

Gene		Primer	Sequence (5’ − 3’)
*Hypoxanthine-guanine phosphoribosyltransferase*	*hHPRT*	Forward	CCTGGCGTCGTGATTAGTGAT
		Reverse	AGACGTTCAGTCCTGTCCATAA
*Μucin 2*	*hMUC2*	Forward	CAGCACCGATTGCTGAGTTG
		Reverse	GCTGGTCATCTCAATGGCAG
*Resistin like beta*	*hRETLNB*	Forward	CACCCAGGAGCTCAGAGATCTAA
		Reverse	ACGGCCCCATCCTGTACA
*Trefoil factor 3*	*hTFF3*	Forward	CATGTCACCCCCAAGGAGTG
		Reverse	AGGTGCATTCTGCTTCCTGC
*Hypoxanthine-guanine phosphoribosyltransferase*	*mHprt*	Forward	AGTCCCAGCGTCGTGATTAG
		Reverse	TTTCCAAATCCTCGGCATAATGA
*Mucin 2*	*mMuc2*	Forward	GAAGCCAGATCCCGAAACCA
		Reverse	CCAGCTTGTGGGTGAGGTAG
*Regenerating islet-derived protein 3 gamma*	*mReg3γ*	Forward	ATGCTTCCCCGTATAACCATCA
		Reverse	GGCCATATCTGCATCATACCAG
*Tumor necrosis factor alpha*	*mTnfα*	Forward	TGGGAGTAGACAAGGTACAACCC
		Reverse	CATCTTCTCAAAATTCGACTGACAA
*Interleukin 1 beta*	*mIL1β*	Forward	TTGACGGACCCCAAAAGATG
		Reverse	AGAAGGTGCTCATGTCCTCA
*Interfefor gamma*	*mIFNγ*	Forward	TGGCTCTGCAGGATT TTCATG
		Reverse	TCAAGTGGCATAGATGTGGAAGAA
*Resistin like beta*	*mRelmβ*	Forward	AAGCCTACACTGTGTTTCCTTTT
		Reverse	GCTTCCTTGATCCTTTGATCCAC
*Trefoil factor 3*	*mTFF3*	Forward	TTGCTGGGTCCTCTGGGATAG
		Reverse	TACACTGCTCCGATGTGACAG
*Interleukin 17*	*mIl17*	Forward	AACACTGAGGCCAAGGACTT
		Reverse	ACCCACCAGCATCTTCTCG
*Interleukin 22*	*mIl22*	Forward	TTGAGGTGTCCAACTTCCAGCA
		Reverse	AGCCGGACGTCTGTGTTGTTA
*Claudin 2*	*Cldn2*	Forward	CAACTGGTGGGCTACATCCTA
		Reverse	CCCTTGGAAAAGCCAACCG

### Inhibitory reagents

Cells were pretreated with an inhibitor of NADPH-oxidases (diphenyleneiodonium chloride- DPI, Sigma Aldrich; 20 µM, 16 h), a noncompetitive dynamin inhibitor of GTPase activity and endocytosis blocker (dynasore, Sigma Aldrich; 80 µM, 30 min), an inhibitor of proteasomes (N-acetylcysteine-NAC, Tocris Bioscience; 0.5 mM, 30 min), a mitochondria-targeted antioxidant (Mito-TEMP, SML0737, Sigma Aldrich), a general inhibitor of protein kinases C (bisindolylmaleimide-BIM-I, Cayman Chemical; 10 µM, 30 min), a selective EGFR inhibitor (AG1478, Tocris Bioscience; 10 µM, 30 min), a selective antagonist of formyl peptide receptor 2 (WRW4, Tocris Bioscience; 10 µM, 30 min), or a selective P2×7 receptor antagonist (A780003, Sigma Aldrich; 10 µM, 30 min). Cells were washed once with PBS before LL-37 stimulation, and supernatants were collected for analysis.

### Immunofluorescence imaging of colons and goblet cells

LS174T cells fixed in 4% paraformaldehyde or frozen murine colon sections were permeabilized (10 min with 0.2% Triton-X100) and washed extensively with PBS-Tween (0.05%) before blocking with 10% normal donkey serum (1 h). Samples were incubated with anti-MUC2 (Santa Cruz Biotechnology; sc -515,106), anti-TFF3 (Santa Cruz Biotechnology; sc -398,651), anti-*E. coli* LPS (SSI Diagnostica; 81449 (SS)), and anti-Ki67 (Abcam, ab16667) antibodies (overnight, 4°C), washed in PBS-Tween and stained with anti-rabbit Alexa Fluor 647, anti-mouse Alexa Fluor 488, or anti-chicken Alexa Fluor 688 (room temperature, 1 h). Samples were washed in PBS-Tween, counterstained with DAPI, and mounted in ProLong Gold Antifade (ThermoFisher). Samples were visualized using a Nikon A1R confocal microscope or an I×71 Olympus fluorescent microscope.

### RNAscope (*in* *situ* hybridization)

*In-situ* hybridization (RNAscope^TM^, Advanced Cell Diagnostics) was conducted to determine ROS activity and goblet cell production using *Duox2*, *Muc2*, and *TFF3* mRNA probes and to detect *C. rodentium* with *espB* mRNA probe. Formalin-fixed distal colons were sectioned (5 μm), baked (60°C, 60 min) to remove paraffin and treated with proteases (30 min, 40°C). Probes for *Duox2*, *Muc2, TFF3* and *espB*, along with positive and negative controls, were pre-heated and hybridized to tissue sections (2 h, 40°C). Signal amplification and washing were performed using the HybEZ Hybridization System. For fluorescence detection, slides were labeled with specific dyes: Alexa-520 (1:500) for *Muc2* and Alexa-647 (1:1500) for *Duox2*, *TFF3* and *espB*. Labeled sections were imaged using a Nikon A1R Confocal Microscope (20X). Signal detection was scored independently and blindly by two evaluators: 0: no staining; 1: 1–3 dots; 2: 4–9 dots; 3: 10–15 dots; and 4: > 15 dots per field of view. For each mouse, a minimum of 5 images from distinct fields of view were analyzed, and the average score per mouse was calculated. This process was repeated for 3–5 mice in each treatment group.

### ROS detection in cells

For isolation of murine colonic epithelial cells, colons were placed in a Petri dish with Hank’s Balanced Salt Solution (HBSS) (14025092, ThermoFisher) without Ca2^+^ and Mg2^+^ containing 10 mM HEPES (HBSS (w/o)). Colons were cleared of feces by holding them with forceps and flushing with HBSS (w/o) using a syringe. After removing residual fat tissue, colons were cut longitudinally and laterally into pieces (0.5 cm length) and transferred into 50 mL tubes containing 20 mL of pre-digestion buffer (1× HBSS (w/o) with 5 mM EDTA, 5% fetal bovine serum (FBS), 1 mM DTT), incubated (20 min, 37°C) under continuous rotation and mixing 10 sec using a vortex mixer, and passed through a 100 µm filter. The proceeding was repeated thrice, and tissues were transferred into a new 50 mL tube containing 20 mL of HBSS (w/o). All the flow through, containing colonic epithelial cells, were combined and centrifuged (300 × *g*, 10 min, at room temperature), and the supernatant was aspirated and discharged; the colonic epithelial cell fractions were resuspended with phenol red-free HBSS for ROS detection.

For determination of ROS, LS174T goblet-like cells or *Camp*^*+/+*^ and *Camp*^*-/-*^ bone marrow-derived macrophages and colonic epithelial cells were plated in black-walled 96-well plates, stimulated with scLL-37, LL-37, phorbol 12-myristate 13-acetate (PMA, 1 µM P1585, Millipore Sigma) or H202 (100 µM, 30 min), and loaded with CM-H2DCFDA (5 µM, C6827, Life Technology) in phenol red-free Hank’s balanced salt solution (HBSS 1402092, Thermofisher) (30 min). Cells were washed twice with HBSS and stimulated with or without H_2_0_2_ (100 µM) in HBSS for 30 minutes. Fluorescence was measured using a microplate reader (Synergy H1, BioTek). The change in fluorescence was reported as an increase in unstimulated cells, with background subtraction from non-dye-loaded cells.

### Bulk RNA sequencing (RNAseq)

Total RNA was extracted from *Camp*^+/+^ and *Camp*^*-/-*^ colons, quantified using (Qubit fluorometer), and the RIN score was assessed using a bioanalyzer (Agilent Bioanalyzer RNA 6000 Nano/Pico Chip). mRNA was isolated and converted into cDNA (NEBNext Poly(A) mRNA Magnetic Isolation; E7490). Libraries were quantified by qPCR (KAPA Library Quantification Kit for Illumina; 07960140001, Roche), and pooled libraries were sequenced on an Illumina NovaSeq 6000 sequencer using a 2 × 50 bp sequencing kit with NovaSeq 6000 SP 100 cycle flow cell reagents. Sequences matching individual transcripts of the *Mus musculus* (GRCm38, Ensembl) transcriptome were quantified using Kallisto (v0.46.1) and aligned using HISAT2 (version 2.2.1) and HTSeq (version 2.0.5). Differential expression analysis was conducted using DESeq2 (v 1.41.2). Pathway enrichment analysis was performed on the top 200 differentially expressed downregulated and upregulated transcripts using the Cytoscape ClueGO v2.5.9 and CluePedia v1.5.9 plugins. The GO databases were used to annotate the enriched pathways. Enrichment was performed using a two-sided hypergeometric test with Bonferroni step-down correction. A κ score of 0.4 was used to define the interaction between terms. Transcripts with a corrected *p* value < 0.05 and absolute log_2_ fold change (Log_2_FC) [Equation]1 were considered differentially expressed. Next, QIAGEN Ingenuity Pathway Analysis (IPA) was utilized to map the genes that exhibited significant absolute log_2_ fold changes (*p* value < 0.05) from the differential expression analyses to the Ingenuity Knowledge Base. Subsequently, IPA core analysis was performed, which identified the known biological pathways associated with the dataset. Those pathways most closely related to ROS production and goblet cell mucin secretion, with z-scores greater than 1 or less than −1, were selected, and the individual genes related to each pathway were further analyzed.

### Microbiota 16S rRNA sequencing

Whole genomic DNA was extracted from stool samples (PowerFecal Pro® DNA Isolation kit, Qiagen 51,804). The 16S rRNA gene V4 region was amplified with primers containing internal barcodes: Forward: AAT-GAT-ACG-GCG-ACC-ACC-GAG-ATC-TAC-AC-barcode-TAT-GGT-AAT-TGT-GTG-CCA-GCM-GCC-GCG-GTA-A, and Reverse: CAA-GCA-GAA-GAC-GGC-ATA-CGA-GAT-barcode-AGT-CAG-TCA-GCC-GGA-CTA-CHV-GGG-TWT-CTA-AT. PCR was performed using KAPA HiFi HotStart master mix (Roche Sequencing) with cycling conditions: 98°C for 2 min, 25 cycles of 98°C for 30 sec, 55°C for 30 sec, 72°C for 20 sec, and final elongation at 72°C for 7 min. PCR products were verified on a 1% agarose gel, purified using NucleoMag NGS Clean-up and Size Select (Macherey-Nagel), and normalized with SequalPrep Normalization Plate (Invitrogen). Amplicons were pooled, and concentration and quality were checked with a Qubit HS DNA kit (Invitrogen) and Tapestation D1000 assay (Agilent). Sequencing was performed on a MiSeq Benchtop DNA sequencer (Illumina) using a V2–500 cycle kit. The pooled library was denatured and prepared with a 5% PhiX Control. A comprehensive analysis of 16S rRNA sequencing was conducted using Microbiome Analyst 2.018. Raw sequence data were processed for quality control, taxonomic assignment, and OTU clustering using the SILVA database (v138.1). Data were analyzed with the ‘DADA2’ package in R for taxonomic classification, alpha diversity, and community structure17 (Analyst2 http://doi.org/10.1093/nar/gkad407). Alpha diversity was visualized using the Marker Data Profiling module, employing Chao1 diversity and t-test statistics. Data were filtered with a 20% low count filter and 10% low variance filter and normalized using total sum scaling. Differences in phyla between the infected groups were analyzed by the Compound Poisson Linear Model (CPLM), including overdispersed count data, such as microbiome amplicon sequence variant data.

### Statistical analysis

Analytical data, represented as histograms, were recorded as mean values, with bars representing standard errors of the mean (SEM) from a minimum of three independent experiments unless stated otherwise. Normality was assessed using the Shapiro-Wilk (Royston) test. All parametric comparisons were performed using a two-sided unpaired Student’s *t*-test or a one-way analysis of variance (ANOVA), with *p* < 0.05. Pairwise comparisons of the means were examined with a *post hoc* Tukey’s test for multiple group comparisons. For non-parametric data, significance was determined using the Kruskal – Walli’s test followed by Dunn’s multiple comparison test. Unless explicitly shown on the graph, a *p-*value was assigned to each group concerning the control group. Statistical significance was set at *p <* 0.05. Statistical analyses were performed using the GraphPad Prism software (Graph Pad 5.0).

## Results

### C. rodentium *infection is more severe in* Camp^−/−^ *littermates*

**Figure 1.**
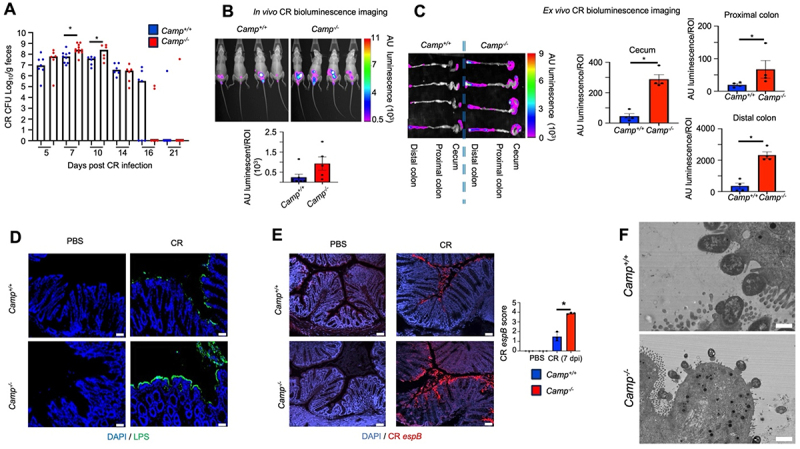
*Camp*^*-/-*^ mice are more susceptible to *C. rodentium* colonization. *Camp*^*+/+*^ and *Camp*^*-/-*^ littermates were infected orally with *C. rodentium* (5 ×10^8^ CFU) or control PBS. (A) fecal shedding of *C. rodentium* was assessed by microbiological culture at the indicated days post-infection (*n* = 6–9 mice/group). (B-C) dissemination of bioluminescent *C. rodentium* with *in vivo* imaging expressed as arbitrary units (AU) of luminescence in (B) the whole body and (C) *ex vivo* excised colons (*n* = 5–7 mice/group; 7- dpi). (D-E) colonization of *C. rodentium* on the colonic epithelium assessed by (D) immunostaining with an anti-LPS antibody (green) (scale bar: 25 µm) and (E) RNAscope staining for *C. rodentium* virulence factor *espB* mRNA (scale bar 50 µm). DAPI (blue) was used as a nuclear marker. Representative images of 3–4 mice/group for 5 fields of view are shown. (F) bacterial adherence and pedestal formation on epithelial cells showed by transmission electron microscopy in distal colonic sections. Scale bar 500 nm. Representative images from 3 mice, 4–5 fields per sample. Data are shown as means ± SEM. *p < 0.05 (two-tailed Student’s t-test or one-way ANOVA *post hoc* Tukey’s test for multiple comparisons) was considered significant.

The kinetics of *C. rodentium* infection showed higher fecal *C. rodentium* shedding in *Camp*^*-/-*^ mice at 7- and 10-days post-infection (dpi) compared to *Camp*^*+/+*^ littermates (Figure 1A). Infections with bioluminescent *C. rodentium* confirmed higher bacterial colonization in *Camp*^*-/-*^ littermates at the peak of infection (7- dpi) by whole-body imaging of the lower abdomen (Figure 1B), and *ex vivo* imaging of the cecum, proximal and distal colons (Figure 1C). Increased susceptibility of *Camp*^*-/-*^ littermates to *C. rodentium* infection was also demonstrated by increased LPS-positive *C. rodentium* on colonic epithelial surfaces (Figure 1D) and abundant expression of *C. rodentium espB* mRNA, a virulence factor necessary for *C. rodentium* colonization and the formation of A/E lesions^50^ (Figure 1E). Pedestal structures compatible with A/E lesions were detected using TEM microscopy but comparably between *C. rodentium*-infected *Camp*^*+/+*^ and *Camp*^*−/−*^ mice (Figure 1F). No direct correlation was found between the frequency of pedestal formations and *espB* expression.

### *Colitis assessment in wild-type and* Camp *knocked-out mice*

**Figure 2.**
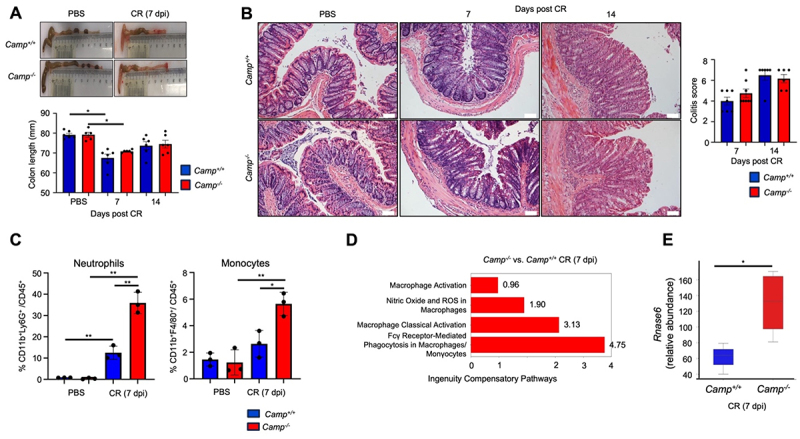
*Camp*^*+/+*^ and *Camp*^*-/-*^ display similar colitis during *C. rodentium* infection. *Camp*^*+/+*^ and *Camp*^*-/-*^ littermates were infected orally with *C. rodentium* (5 ×10^8^ CFU) or PBS. (A) Colon length was determined at 7- and 14- dpi with *C. rodentium* (*n* = 5–6/group). (B) Microphotographs of distal colons at the indicated days post-infection and histopathological scores (hematoxylin & eosin) (*n* = 6–8 mice/group). Scale bar 50 µm. (C) Flow cytometric analysis of colonic lamina propria immune cells showing the percentage of neutrophils and macrophages from total CD45^+^ cells at *C. rodentium* infection peak (7- dpi). (D) Ingenuity canonical bulk-RNA-seq pathways in *Camp*^*+/+*^ and *Camp*^*-/-*^ colons at *C. rodentium* infection peak (7- dpi). The bar graph shows significant pathways with positive and negative Fold changes, indicating upregulation or downregulation of various signalling pathways and detoxification processes. (E) Gene relative abundance of *Rnase6*, an epithelial-derived antimicrobial peptide member of the RNase a superfamily. Data are shown as means ± SEM. *p < 0.05 and ** *p* < 0.01 (two-tailed Student’s t-test or one-way ANOVA *post hoc* Tukey’s test for multiple comparisons) were considered significant.

Although *Camp*^−/−^ were more heavily infected with *C. rodentium*, colitis was similar between the littermates at 7- and 14- dpi as determined by comparable colon shortening (Figure 2A) and histopathological scores for crypt hyperplasia and leukocyte infiltration in the lamina propria (Figure 2B). Likewise, both genotypes displayed similar transcriptional expression of pro-inflammatory cytokines *Il17, Il22*, and *Ifng*, and goblet cell-specific *Muc2*, *Tff3*, and *Retnlβ* components, although *Camp*^*-/-*^ littermates showed significantly higher *Il1b* and *Tnfa* gene expression at 7- dpi (Figure S1A). The number of infiltrating neutrophils and monocytes in the lamina propria was also higher in *C. rodentium*-infected *Camp*^*-/-*^ colons compared to their *Camp*^*+/+*^ counterparts (Figure 2C). Furthermore, we demonstrated in *Camp*^*−/−*^ colons 7- dpi with *C. rodentium* an upregulation of genes related to macrophage-associated pathways, including classical activation, production of nitric oxide and reactive oxygen species, Fcγ receptor-mediated phagocytosis, and enhanced type I interferon signaling (Figure 2D). This bulk-RNA-seq analysis also showed an increased abundance of *Rnase6*, a gene encoding an epithelial-derived antimicrobial peptide, RNAse6, in *Camp^−^/^−^* colons (Figure 2E).

Despite similar levels of bacterial translocation to the liver and spleen (Figure S1B), the integrity of the epithelial barrier and tight junctions in *C. rodentium*-infected *Camp*^*− /−*^ colons was compromised compared to their *Camp*^+/+^ counterparts as indicated by increased permeability to 4-kDa FITC dextran and decreased expression of the *Claudin-2* gene at 7- dpi with *C. rodentium* (Figure S1C). These results show that *Camp*^*-/-*^ were more susceptible to *C. rodentium* infection, with increased epithelial colonization and bacterial shedding. However, both littermates cleared the infection and mounted a similar pro-inflammatory response.

### *Microbial diversity in* Camp^−/−^ *littermates is less abundant basally and in response to* C. rodentium *infection*

**Figure 3.**
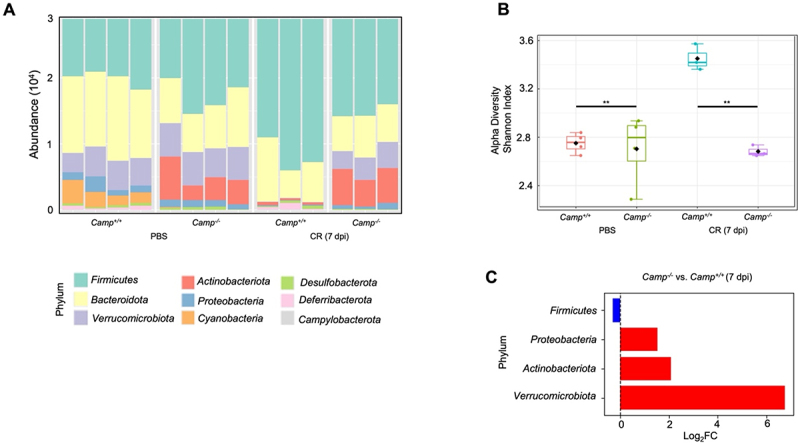
Changes in the fecal microbiota during *C. rodentium* infection. (A) Bar plot displaying the relative abundance of bacterial taxa at the phylum level across different samples. Each bar represents a sample, and colours represent different bacterial species. (B) Alpha diversity analysis based on the Shannon diversity index. Each dot represents a sample. *p < 0.05 (two-tailed Student’s t-test or one-way ANOVA post hoc Tukey’s test for multiple comparisons) was considered significant. (C) Differential abundance analysis comparing the relative abundance between *Camp*^*-/-*^ and *Camp*^*+/+*^ groups at 7- dpi, displayed as Log_2_ Fold change (Log_2_FC).

Baseline microbial composition in *Camp*^*-/-*^ littermates showed a lack of *Cyanobacteria* and abundance of *Actinobacteriota* compared to their *Camp*^*+/+*^ counterparts (Figure 3A), but alpha diversity was similar between groups (Figure 3B). At peak *C. rodentium* infection (7- dpi), the microbiota in *Camp*^*+/+*^ showed higher levels of *Actinobacteriota* and *Fimicutes* and lower levels of *Cyanobacteria, Verrucomicrobiota*, and *Proteobacteria* compared with uninfected *Camp*^*+/+*^ littermates (Figure 3A). Remarkably, the microbial composition in *Camp*^*-/-*^ littermates infected with *C. rodentium* resembled that of uninfected *Camp*^*-/-*^ controls (Figure 3A). As expected, *C. rodentium*-infected *Camp*^*+/+*^ showed increased alpha diversity (Shannon index) compared with a less noticeable microbiota shift in *Camp*^*-/-*^ littermates in response to *C. rodentium* (Figure 3B). Microbial phyla comparison revealed significant differences between *Camp*^*+/+*^ and *Camp*^*-/-*^ littermates infected with *C. rodentium* (Figure 3C). *Verrucomicrobiota* increased the most (6.8-fold) in *C. rodentium*-infected *Camp*^*-/-*^ as compared to *Camp*^*+/+*^ littermates (*p* = 8.57 × 10^−8^), followed by *Actinobacteriota* (2-fold) (*p* = 3.06 × 10^−5^), and *Proteobacteria* (1.5-fold) (*p* = 0.0112). Conversely, *Firmicutes* were reduced in *C. rodentium*-infected *Camp*^*-/-*^ littermates (−0.3-fold; *p* = 0.0137) (Figure 3C). These results underscore notable shifts in microbial phyla associated with the *Camp*^*-/-*^ phenotype.

### Camp^−/−^ *littermates display defective goblet cell mucus secretion during* C. rodentium *infection*

**Figure 4.**
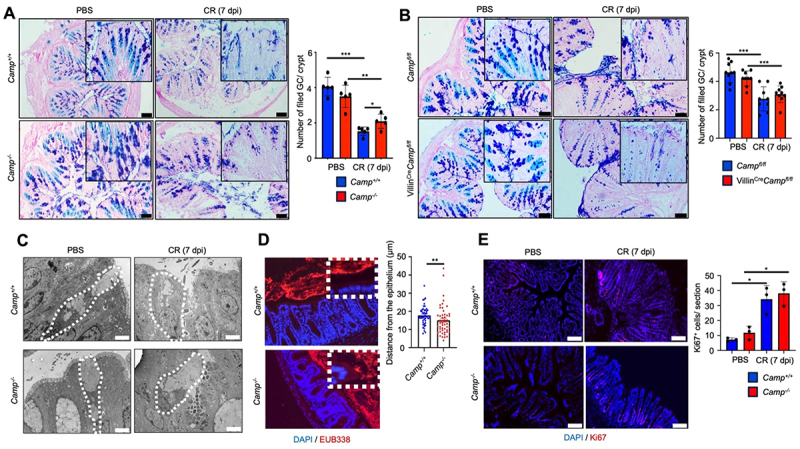
*Camp*^*-/-*^ littermates display dysfunctional goblet cell mucus secretion. (A-B) identification and quantification of goblet cells by Alcian blue and PAS staining in distal colons of (A) *Camp*^*+/+*^ and *Camp*^*-/-*^ littermates and (B) *Camp*^*fl/fl*^ and *Villin*^*Cre*^*Camp*^*fl/fl*^ infected orally with *C. rodentium* (5 ×10^8^ CFU) or control PBS. Goblet cells were quantified at *C. rodentium* infection peak (7- dpi) as the number of Alcian blue full-filled goblet cells per crypt (n 4–5 mice/group, with at least 6 crypts counted per mouse). Scale bar 20 µm. (C) transmission electron microscopy of goblet cells in the colon and quantification of mucin granules per goblet cell in controls (*n* = 3 mice/group, with at least 5 goblet cells imaged per mouse). Scale bar 2 µm. (D) FISH of uninfected colons using a pan-bacterial probe (EUB338-red) and DAPI (blue) to visualize the separation of the microbiota and epithelium (*n* = 3 mice/group with at least 13 measurements taken at random locations along the colonic cross-section). Scale bar 50 µm. Data are shown as individual measurements. (E) immunofluorescence Ki67 staining for epithelial proliferation (red). Three mice per group for 5 different fields of view were captured. The average numbers of Ki67^+^ cells were measured using ImageJ. Scale bar 10 µm. Data are shown as means ± SEM. *p < 0.05, **p < 0.01, and ***p < 0.001 (two-tailed Student’s t-test or one-way ANOVA *post hoc* Tukey’s test for multiple comparisons) were considered significant.

To decipher how endogenous cathelicidin modulates *C. rodentium* infection, we focused on the colonic mucus layer, as it is essential for innate host defense against *C. rodentium*^2^ and is thinner and more easily penetrated by *E. coli* O157:H7 in *Camp*^*-/-*^ littermates.^42^ We quantified the number of mucus-filled goblet cells by measuring the number of Alcian blue/Periodic acid Schiff (PAS)-stained positive cells per crypt in the distal colon. Similar numbers of mucus-filled goblet cells were observed for *Camp*^*+/+*^ and *Camp*^*-/-*^ littermates at basal levels (Figure 4A). However, at *C. rodentium* infection peak (7- dpi), there were fewer mucus-filled goblet cells in *Camp*^*+/+*^ as compared to *Camp*^*-/-*^ littermates (Figure 4A). To determine whether epithelial-produced cathelicidin would be responsible for the increase in mucus-filled goblet cells during *C. rodentium* infection, we challenged *C. rodentium* mice lacking cathelicidin in intestinal epithelial cells (*Villin*^*Cre*^*Camp*^*fl/fl*^). *Villin*^*Cre*^*Camp*^*fl/fl*^ mice infected with *C. rodentium* expressed reduced numbers of mucus-filled goblet cells in the distal colon compared to *Camp*^*fl/fl*^ counterparts (Figure 4B) with similar bacterial shedding (Figure S1D). These findings indicate that non-epithelial sources of cathelicidin (e.g., neutrophils) were likely involved in the phenotype of mucus-filled goblet cells in *Camp*^*-/-*^ littermates infected with *C. rodentium*. By transmission electron microscopy (TEM), goblet cells in the colons of *Camp*^*+/+*^ littermates were shrunken and contained few or no granules at peak *C. rodentium* infection (7- dpi) (Figure 4C). In stark contrast, goblet cells in *Camp*^*-/-*^ littermates were swollen and filled with mucus granules in response to *C. rodentium* (Figure 4C). Based on these findings, we assess if bacteria were near the mucosal surface by fluorescent *in situ* hybridization (FISH) using a pan-bacterial probe (EUB338). *Camp*^*-/-*^ showed microbiota close to the colonic surface as compared to *Camp*^*+/+*^ littermates (Figure 4D). The response in epithelial cell proliferation was similar between *Camp*^*+/+*^ and *Camp*^*-/-*^ colon, showing comparable increased number of Ki67^+^ cells at peak of *C. rodentium* infection (7- dpi) (Figure 4E).

### *Cathelicidin modulates the secretion of goblet cell innate effectors during* C. rodentium *infection*

**Figure 5.**
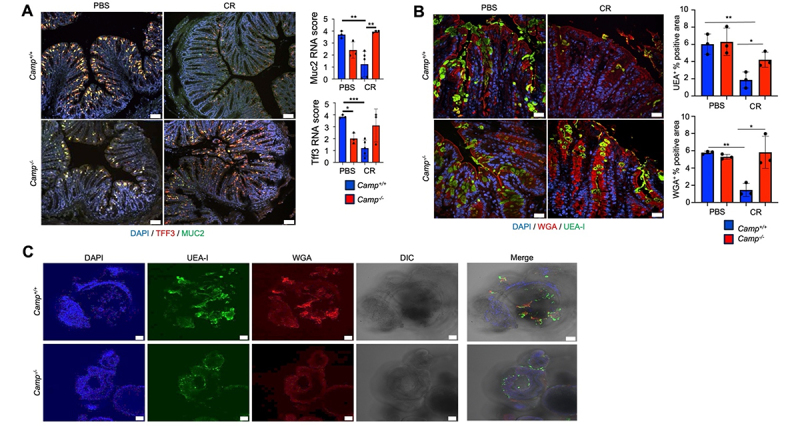
*Camp*^*-/-*^ littermates have an altered colonic mucus barrier. *Camp*^*+/+*^ and *Camp*^*-/-*^ littermates were infected orally with *C. rodentium* (5 ×10^8^ CFU) or control PBS, and distal colons were collected and analyzed at infection peak (7- dpi). (A) RNAscope assay staining for Muc2 (green) and Tff3 (red) mRNAs (*n* = 3 mice per group for a total of 5 different fields of view). Scale bar 50 µm. Scored was adapted from the ACD RNAscope manual. (B) immunofluorescence glycan staining for fucose (UEA-I; green) and N-acetylglucosamine sialic acid (WGA; red). Three mice per group for 5 different fields of view were captured, and the percent positive areas were measured using ImageJ. Scale bar 25 µm. (C) confocal microphotographs of colonoid spheroids derived from *Camp*^*+/+*^ and *Camp*^*-/-*^ littermates stained for fucose (UEA-I^+^; green) and sialic acid (WGA^+^; red) glycosylated mucin using DAPI to stain nuclei (representative images of *n* = 3 individual imaging experiments shown). Scale bar 50 µm. Data are shown as means ± SEM. *p < 0.05, **p < 0.01, and ***p < 0.001 (two-tailed Student’s t-test or one-way ANOVA *post hoc* Tukey’s test for multiple comparisons) were considered significant.

Goblet cells in the colon secrete MUC2 mucin and peptides, including TFF3,^9,51^ and here we determined whether cathelicidin deficiency affected the expression of several goblet cell components. Basally, *Camp*^*+/+*^ expressed slightly higher levels of *Muc2*- and *Tff3*- mRNA in the upper third crypts as compared to *Camp*^*-/-*^ littermates (Figure 5A). However, at peak *C. rodentium* infection, *Camp*^*+/+*^ goblet cells showed marked reduction in *Muc2* and *Tff3* mRNA expression whereas the expression in *Camp*^*-/-*^ goblet cells was unchanged (Figure 5A).

We next studied the impact of cathelicidin on Muc2 posttranslational *O-*linked glycosylation.^52^ *Camp*^*+/+*^ and *Camp*^*-/-*^ littermates showed comparable glycosylated mucin at homeostasis with presence of N-acetyl-D-glucosamine and sialic acid in the upper third of the crypts as determined by lectin WGA (Wheat germ agglutin; WGA^+^) and fucose (*Ulex Europaeus* agglutinin; UEA-I^+^) staining (Figure 5B). At peak *C. rodentium* infection, *Camp*^*+/+*^ goblet cells displayed significantly less WGA^+^ and UEA-I^+^ mucin (Figure 5B). In contrast, *Camp*^*-/-*^ goblet cells in response to *C. rodentium* showed swollen WGA^+^/UEA-I^+^ goblet cells in the upper third of the crypts (Figure 5B). To confirm that colonic *Camp*^*-/-*^ goblet cells accumulate glycosylated mucin and exhibit impaired granule exocytosis, intestinal epithelial spheroids were generated from freshly isolated colonic crypts from *Camp*^*+/+*^ and *Camp*^*-/-*^ littermates and cultured in the presence of the Notch inhibitor DAPT to stimulate goblet cell differentiation.^53^ Visually, *Camp*^*+/+*^ colonic spheroids secreted WGA^+^ and UEA-I^+^ thick mucus strands in the lumen (Figure 5C; Videos S1–2), whereas *Camp*^*-/-*^ colonic spheroids secreted less WGA^+^ and UEA-I^+^ mucin and retained mucus within goblet cells (Figure 5C; Videos S1–2).

### Human cathelicidin stimulates TFF3 and RELMβ secretion from LS174T goblet-like cells

**Figure 6.**
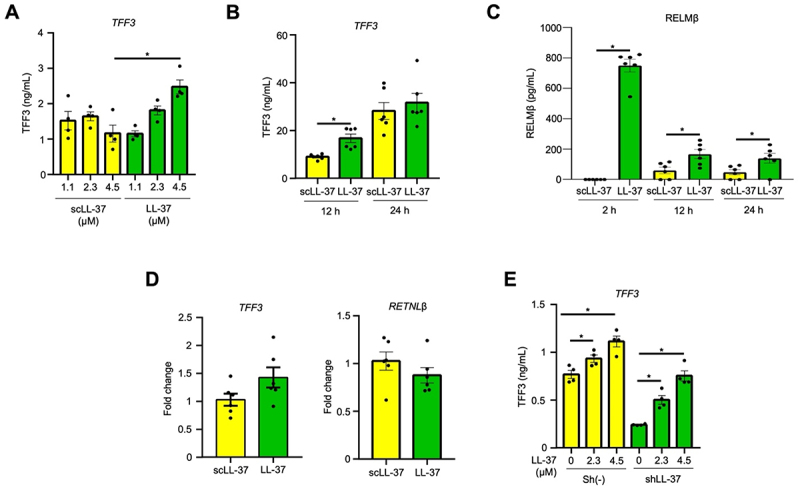
LL-37 stimulates the secretion of TFF3 and RELMβ from colonic goblet-like cells. Human colonic epithelial-like goblet LS174T cells were stimulated with human cathelicidin LL-37 or a scrambled peptide (scLL-37) (4.5 µM; 2 h). (A-B) quantification of secreted TFF3 in the supernatant of LS174T cells stimulated with (A) the indicated concentrations of LL-37 or scLL-37 for 2 h and (B) a fixed dose of LL-37 and scLL-37 (4.5 µM) for 12 and 24 h. (C) secreted RELMβ in the supernatant of LS174T cells stimulated with LL-37 or scLL-37 (4.5 µM;) for 2 h, 12 h and 24 h. (D) TFF3 and RETNLB gene expression in LS174T cells stimulated with scLL-37 or LL-37 (4.5 µM) for 2 h. (E) quantification of secreted TFF3 in LS174T cells following silencing of the *CAMP* gene (shLL-37) or vector (control, sh(-)) cells stimulated with increasing concentrations of LL-37. For all experiments, *n* = 4–6. Data are shown as means ± SEM. *p < 0.05 (two-tailed Student’s t-test or one-way ANOVA *post hoc* Tukey’s test for multiple comparisons) was considered significant.

To determine whether cathelicidins can directly modulate goblet cell functions, we used human LS174T goblet-like cells, a well-established model to study goblet cell mucus secretion.^54^ LS174T cells basally expressed MUC2 and TFF3 (Fig. S2A) and were not cytotoxic to exogenously added human cathelicidin LL-37 up to 4.5 µM (Fig. S2B). These low and non-cytotoxic concentrations of LL-37 stimulated the secretion of TFF3 as early as 2 h (Figure 6A), which peaked up to 24 h post-stimulation (Figure 6B). A scrambled peptide (scLL-37) did not stimulate TFF3 (Figure 6A,B). LL-37 also stimulated the secretion of RELM-β, which peaked as early as 2 h post-stimulation and remained high up to 12 and 24 h (Figure 6C) compared to scLL-37. The LL-37-induced secretion of TFF3 and RELM-β was posttranscriptional, as *TFF3* and *RETNLβ* mRNA expression did not change following stimulation with LL-37 (Figure 6D). To determine whether endogenous cathelicidin could similarly regulate TFF3 secretion, the human cathelicidin gene (*CAMP*) was silenced in LS174T cells. Sham sh(-) LS174T cells produced measurable basal levels of endogenous LL-37 (~10–11 ng/mL), which were significantly decreased in cathelicidin-silenced cells (shLL-37) (Fig. S2C). As predicted, exogenous LL-37 stimulated the secretion of TFF3 in sh (-) cells and lower levels in shLL-37 cells (Figure 6E). These results demonstrate that both exogenous and endogenous cathelicidin regulate the secretion of TFF3 and RELMβ in LS174T goblet-like cells.

### *Canonical mucin-producing goblet cells are depleted* in Camp^−/−^ *littermates during* C. rodentium *infection*

**Figure 7.**
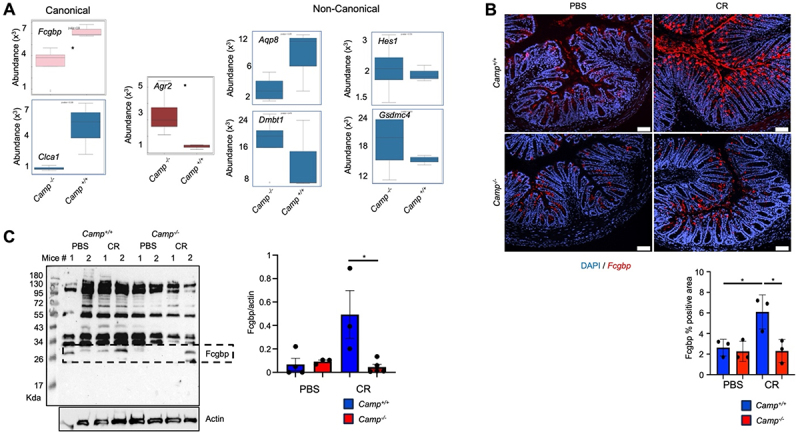
Alterations in Fcgbp in canonical *Camp*^*-/-*^ goblet cells. *Camp*^*+/+*^ and *Camp*^*-/-*^ littermates were infected orally with *C. rodentium* (5 ×10^8^ CFU) or control PBS, and distal colons were collected and analyzed at *C. rodentium* infection peak (7- dpi). (A) gene abundance of goblet cell canonical *Fcgbp* and *Clca1*, *Agr2*, and non-canonical *Aqp8*, *Hes1*, *Dmbt1*, *Gsdmc4* makers in *Camp*^*+/+*^ and *Camp*^*-/-*^ littermates infected with *C. rodentium* based on bulk RNA analysis. (B) RNAscope assay staining for Fcgbp (red) mRNAs (*n* = 3 mice per group for 5 fields of view). Scale bar 50 µm. Scoring was adapted from the ACD RNAscope manual. (C) Western blotting analysis for Fcgbp protein in whole colons (*n* = 3–4 mice per group). Quantification and finding the ratio of Fcgbp over β-actin was done through ImageJ. For all experiments, *n* = 3–6. Data are shown as means ± SEM. *p < 0.05 (two-tailed Student’s t-test or one-way ANOVA *post hoc* Tukey’s test for multiple comparisons) was considered significant.

Deficiencies in Muc2 mucin render mice more vulnerable to *C. rodentium* colonization and colitis.^2^ In *Camp*^*-/-*^ littermates, we observed subtle alterations in the mucus barrier with bloated mucin-filled goblet cells associated with higher *C. rodentium* shedding. Based on these findings, we investigated the characteristics of the goblet cells in *Camp* littermates during *C. rodentium* infection. We focused on “canonical” mucin-producing goblet cells rich in Fc fragment of IgG-binding protein (*Fcgbp*) and calcium-activated chloride channel accessory 1 (*Clca1*).^18^ Bulk RNA-seq data derived from colons at peak *C. rodentium* infection showed significantly augmented *Fcgbp* mRNA and slightly high *Clca1* mRNA expression (*p* = 0.1) in *Camp*^*+/+*^ as compared with *Camp*^*-/-*^ counterparts (Figure 7A,Table 2). At the same time, goblet cell-specific gene anterior gradient protein (*Agr*) 2, related to mucin folding and biosynthesis,^55^ was comparatively lower in *Camp*^*+/+*^ colons (Figure 7A,Table 2). There were no significant changes between *C. rodentium*-infected *Camp*^*+/+*^ and *Camp*^*-/-*^ colons in the non-canonical goblet cell makers, *Aqp8*, *Hes1*, *Dmbt1*, and *Gsdmc4* (Figure 7A). Deficient Fcgbp proteins in goblet cells in *Camp*^*-/-*^ colons during infection was confirmed by immunofluorescence and western blotting (Figure 7B,C). At peak *C. rodentium* infection, *Camp*^*+/+*^ colonic mucosa showed higher numbers of *Fcgbp*^*+*^ goblet cells in the upper third of crypts and abundant secreted *Fcgbp*^*+*^mucus (Figure 7B). In contrast, *Fcgbp*^*+*^ goblet cells in *C. rodentium*-infected *Camp*^*-/-*^ colons were similar to uninfected controls (Figure 7B). Increased Fcgbp protein expression in *Camp*^*+/+*^ colons was confirmed by immunoblotting as compared with *Camp*^*-/-*^ littermates (Figure 7C). Taken together, these findings demonstrate that *Camp*^*-/-*^ were deficient in canonical mucin-secreting *Fcgbp*^*+*^ goblet cells during *C. rodentium* colitis.

**Table 2.** Proteins downregulated in *Camp* ^*-/-*^ littermates after 7 days post *C. rodentium* infection.

Protein	*Gene*	Log 2 value* (*Camp* ^*-/-*^*: Camp* ^*+/+*^)	Biological process (GO)
Mucin 2	*Muc2*	−4.8	Major constituent of large polymeric glycoprotein networks
Zymogen granule membrane protein 16	*Zg16*	−3.34	Binds Gram +ve bacteria and aggregates them.
Calcium-activated activated channel regulator 1	*Clca1*	−2.73	Mucus expansion.
Anterior gradient protein 2 homolog	*Agr2*	−1.54	Folding, trafficking and assembly on cysteine rich Muc2
Fc fragment of IgG-binding protein	*Fcgbp*	−1.19	Gel-forming mucus as an innate host defense barrier at mucosal surfaces.

*Changes in abundance of proteins as Log2 means Log2 values < 0 represent downregulation in *Camp* ^*-/-*^ compared with *Camp* ^*+/+*^ littermates.

### Camp^−/−^ *littermates are deficient in ROS biosynthesis, contributing to defective mucin secretion during* C. rodentium *infection*

**Figure 8.**
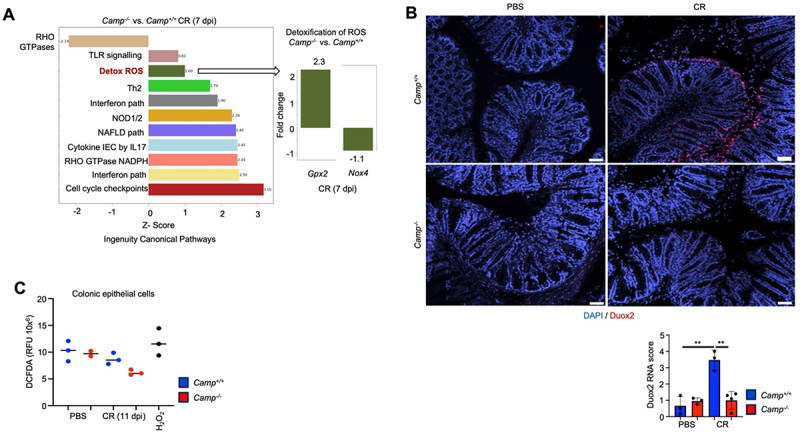
*Camp*^*-/-*^ littermates show distinctive transcriptional profiling associated with impaired reactive oxygen species (ROS) synthesis. (A) Ingenuity canonical pathways in *Camp*^*+/+*^ and *Camp*^*-/-*^ littermates at *C. rodentium* infection peak (7- dpi). The bar graph shows significant pathways with positive and negative Fold changes, indicating upregulation or downregulation of various signalling pathways and detoxification processes. The inset bar graph highlights the Fold change in the expression of *Gpx2* and *Nox4* genes related to ROS detoxification in *Camp*^*+/+*^ and *Camp*^*-/-*^ littermates. (B) RNAscope assay staining for Duox 2 (red) mRNAs (*n* = 3 mice per group for 5 fields of view). Scale bar 50 µm. Scoring was adapted from the ACD RNAscope manual. (C) ROS levels measured by DCFDA fluorescence in colonic epithelial cells of *Camp*^*+/+*^ and *Camp*^*-/-*^ littermates. For all experiments, *n* = 3–6. Data are shown as means ± SEM. ** *p* < 0.01 (two-tailed Student’s t-test or one-way ANOVA *post hoc* Tukey’s test for multiple comparisons) was considered significant.

To shed light on the putative mechanism by which endogenous cathelicidin regulates mucin secretion in colonic goblet cells in response to infection, we focused on ROS that regulates mucin secretion in the colon.^20^ By bulk RNA-seq transcript analysis, there was an upregulation of genes associated with detoxification of ROS during peak *C. rodentium* infection in *Camp*^*-/-*^ compared to *Camp*^*+/+*^ littermates (Figure 8A; Table S1). Two genes contributed to a net positive ROS balance in *Camp*^*+/+*^ littermates: antioxidant glutathione peroxidase 2 (Gpx*2*), which was increased in *Camp*^*-/-*^, and NADPH oxidase Nox4, which was decreased in *Camp*^*-/-*^ colons (Figure 8A). High ROS in *Camp*^*+/+*^ during infection was confirmed by elevated levels of dual oxidase 2 (*Duox2*) mRNA, an essential source of hydrogen peroxide, whereas *C. rodentium*-infected *Camp*^*-/-*^ colonic epithelium showed minimal *Duox2* expression (Figure 8B). The colonic epithelium was a significant source of ROS, as colonic epithelia extracted from *Camp*^*-/-*^ infected with *C. rodentium* at 11- dpi released less ROS than those from *Camp*^*+/+*^ littermates (Figure 8C). These results suggest that *Camp*^*+/+*^ littermates produced higher ROS and canonical pathways associated with less detoxification in response to infection. Such impaired ROS production at the colonic mucosa in *Camp*^*-/-*^ littermates seems to lead to aberrant mucin secretion in response to *C. rodentium*.

### Cathelicidin stimulates the secretion of TFF3 from goblet cells via ROS

**Figure 9.**
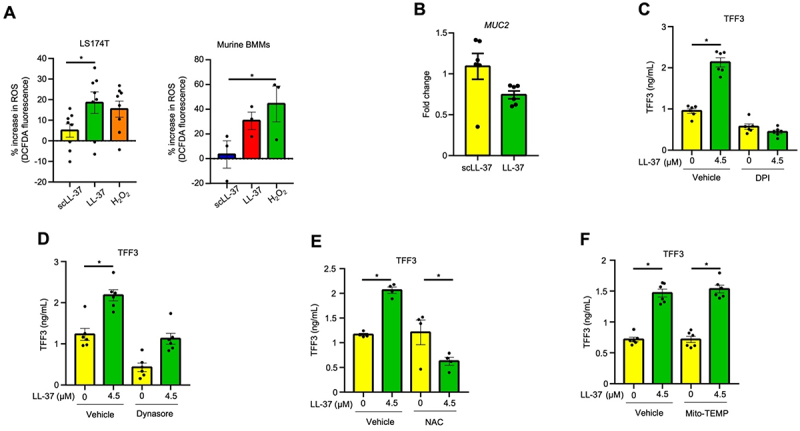
LL-37-induced TFF3 secretion requires reactive oxygen species (ROS) production. (A) ROS production in human LS174T colonic goblet cells and WT murine bone marrow-derived macrophages stimulated with LL-37, scramble peptide (scLL-37) (4.5 µM) or the positive control, hydrogen peroxide (H_2_0_2_; 100 µM) for 30 min. Cells were exposed to a general oxidative stress indicator (CM-H2DCFDA), and ROS production was measured using a fluorescent plate reader. Data are represented as a % increase relative to unstimulated cells ( = 8 for LS174T cells and *n* = 3 for murine BMMs). (B) *MUC2* gene expression in LS174T cells stimulated with scLL-37 or LL-37 (4.5 µM) for 2 h. (C-E) quantification of secreted TFF3 in LS174T cells pretreated (30 min) with (C) an NADPH oxidase inhibitor (diphenyleneidodonium, DPI), (D) an endocytosis inhibitor (dynasore), (E) a scavenger for intracellular ROS (N-acetyl-l-cysteine, NAC), and (F) a mitochondria-specific ROS scavenger (Mito-TEMPO) followed by stimulation with LL-37 (4.5 µM; 2 h) (*n* = 4–6/group). Data are shown as means ± SEM. *p < 0.05 (one-way ANOVA *post hoc* Tukey’s test for multiple comparisons) was considered significant.

**Figure 10.**
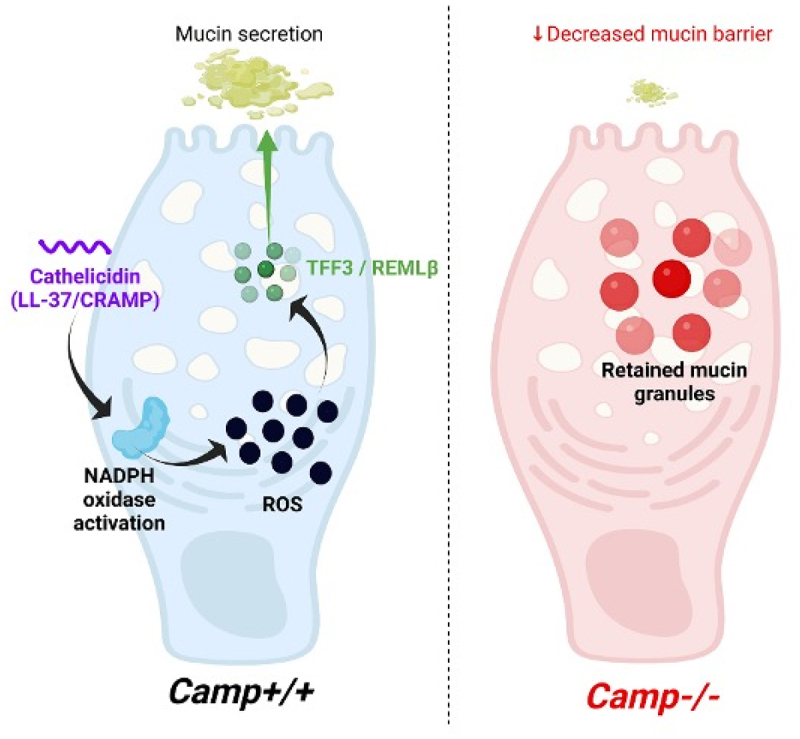
A theoretical scheme of functions elicited by cathelicidins to promote mucus secretion in colonic goblet cells during infection. During intestinal infection with attaching/effacing *citrobacter rodentium* enteropathogen, cathelicidin released by non-epithelial cells, likely derived from infiltration neutrophils, activates NADPH oxidases and the production of reactive oxygen species (ROS). This ROS promotes the release of mucin granules and goblet cell-associated peptides, trefoil factor 3 (TFF3) and resistin-like molecule β (RELMβ). This enhancement in the colonic mucin barrier is implicated in lesser bacterial shedding at the peak of infection.

As ROS mediates mucus secretion in goblet cells,^19,20^ and cathelicidin stimulates ROS in leukocytes,^56,57^ we determined whether cathelicidin can modulate MUC2 and TFF3 secretion in goblet cells by inducing ROS. Synthetic LL-37, but not scLL-37, stimulated intracellular ROS production in LS174T goblet-like cells and bone marrow-derived murine macrophages (BMMs) (Figure 9A). While LL-37 did not affect MUC2 mRNA expression (Figure 9B), it stimulated TFF3 secretion via ROS that was inhibited with the NADPH oxidase inhibitor, diphenyleneiodonium (DPI; Figure 9C). Moreover, we interrogated the role of NADPH oxidases, which are endocytosed from the plasma membrane during ROS synthesis in leukocytes and goblet cells.^20,58^ We observed that inhibition of endocytosis with dynasore also inhibited LL-37-induced TFF3 secretion (Figure 9D). Inhibition of intracellular ROS by *N*-acetylcysteine (NAC), which interacts directly with ROS, likewise abolished TFF3 secretion (Figure 9E). The source of ROS was unlikely from mitochondria as mitochondria-specific antioxidant ROS scavenger (Mito-TEMP) did not affect LL-37-induced TFF3 secretion (Figure 9F). In the search for an LL-37 receptor that mediates ROS and downstream TFF3 secretion, we explored the involvement of cellular receptors previously reported to modulate cathelicidin functions using selective chemical inhibitors or antagonists. These include epidermal growth factor receptor (EGFR)^23,59^ with an EGFR inhibitor (AG1478), P2X purinoceptor 7 (P2X7)^60^ with a P2×7receptor antagonist (A780003), and N-formyl peptide receptor 2 (FPR2)^61^ with an FPR2 antagonist (WRW4). None of these molecules affected LL-37-induced TFF3 in LS174 cells (Fig. S3A-C). These data reveal that cathelicidin is a stimulator of ROS via NADPH, which induces the secretion of mucin and other goblet cell proteins (Figure 10).

## Discussion

This study investigated how cathelicidin regulates intestinal barrier function during *C. rodentium* infection and uncover an innate role for this peptide in regulating mucin secretion and associated peptides, critical in the mucus barrier, excluding luminal microbiota from the epithelia^62^ and limiting colonization of *C. rodentium*.^2^ Mice deficient in cathelicidin (*Camp*^*-/-*^) exhibited increased *C. rodentium* colonization and *espb mRNA* expression near the mucosal surface, along with pedestal formation compatible with A/E lesions. Studies *in vitro* using human LS174T goblet-like cells and murine colonoids confirmed that *Camp*^*-/-*^ secreted less mucus with an altered glycosylation profile associated with decreased ROS biosynthesis in the colon.

The findings here highlight that cathelicidin regulates mucus secretion to establish a functional mucus layer during *C. rodentium* infection and has potential roles in mucus exocytosis in colonic goblet cells. This role of cathelicidin in the secretion of intestinal mucin was noticeable in *Camp*^*−/−*^ mice infected with *C. rodentium*, which showed a thinner or discontinuous mucus layer, along with goblet cells exhibiting swollen morphology and an accumulation of mucus granules. Deficiency in cathelicidin also affected TFF3 and RELMβ secretions. TFF3 is essential for epithelial repair^8,63–65^ and healing in DSS colitis^65^ and swiftly responds to inflammation,^66^ preventing epithelial cell apoptosis.^13,14,67^ RELMβ promotes epithelial migration and prevents apoptosis,^10^ while its deficiency (*Retlnb*^*-/-*^) renders mice susceptible to *C. rodentium* infection.^17^ RELMβ secretion by goblet cells is also chemoattractant for CD4^+^ T cells^17,68^ and induces REGIIIβ, a lectin that kills Gram-positive bacteria.^69^ Considering the higher *C. rodentium* burden in *Camp*^*-/-*^ at peak infection, the timely release of RELMβ stimulated by cathelicidin may account for early pathogen clearance. Mechanisms by which cathelicidins regulate mucin exocytosis may be diverse. First, the impact of LL-37 on the secretion of goblet cell-associated proteins occurred post-transcriptionally, as LL-37 did not upregulate *MUC2*, *TFF3*, and *RELMβ* mRNA in LS174T goblet-like cells following stimulation. Other studies have shown that LL-37 upregulates *MUC2* gene expression in colonic epithelial (HT-29) cells^43,44^ and *MUC5AC* in airway epithelial (NCI-H292) cells.^59^ Then, pathways by which cathelicidin regulates mucus and posttranscriptional secretion of goblet cell-associated peptides TFF3 and RELMβ may include SNARE protein-mediated exocytosis, where VAMP8 coordinates the fusion of mucin granules with the plasma membrane, facilitating the expulsion of MUC2 mucin into the intestinal lumen.^70^ Likewise, cathelicidin could regulate the release of intracellular Ca^2^ -dependent mucins, which is commonly triggered by mucin-secretagogue agents such as acetylcholine and histamine.^71^ It is noted that the defect in the intestinal mucin barrier in *Camp*^*−/−*^ mice infected with *C. rodentium* was not attributable to different epithelial proliferation.

Cumulative evidence shows that goblet cells are a heterogeneous population with functions depending on their location along the crypt.^18,19,72^ For instance, canonical goblet cells produce typical goblet cell components such as Muc2, Fcgbp, and Clca1, while non-canonical goblet cells display a mixed profile with features more typical of enterocytes.^18^ *Camp*^*-/-*^ littermates showed lower canonical *Fcgbp* and *Clca1* expression during *C. rodentium* infection, denoting that endogenous cathelicidin modulates the goblet cells’ primary function to produce mucin. Fcgbp and MUC2 are colocalized within mucin granules, forming a structured mucus barrier that limits microbial access to the epithelium.^73^ Another substantial part of FCGBP is diffusely distributed in the cytoplasm of goblet cells, dissociated from the theca,^73^ and secreted independently of MUC2, stabilized by disulfide bonds.^74^ *Fcgbp*^*+*^ goblet cells represent a distinct subset enriched in inflamed colons.^73^ Our observed deficiency in canonical mucin-secreting *Fcgbp*^*+*^ goblet cells in *Camp*^*-/-*^ mice infected with *C. rodentium* would thus compromise the structural integrity and organization of the mucus layer, resulting in a thinner or less cohesive barrier and increased susceptibility to epithelial colonization. Interestingly, goblet cells at the neck of the crypts are key sensing ligands for TLRs (e.g., TLR4).^19^ Since cathelicidin promotes TLR4 signaling in the colon,^23^ this peptide might promote goblet cell sensing capacity and mucin release at the crypt entrance.

Transcriptomic analyses revealed a negative ROS balance in *Camp*^*-/-*^ littermates colons during infection, with lesser synthesis of key ROS enzymes, such as NADPH oxidases (Nox4 and Duox2), and increased ROS detoxification. Similar defective ROS production was observed in *Camp*^*-/-*^ bone marrow-derived macrophages, which had decreased ROS synthesis than their wild-type counterparts. Production of ROS in the gut by macrophages may be significant in *C. rodentium* colitis.^75^ ROS regulates the exocytosis of mucin granules in goblet cells.^19,20^ Thus, cathelicidin regulating ROS production could induce mucin secretion by goblet cells.

The source of cathelicidin in *C. rodentium* infection modulating colonic mucin dynamics is most likely derived from non-epithelial cells, primarily neutrophils, since epithelial-specific deletion of cathelicidin did not lead to goblet cell swelling or mucus accumulation. An abundance of cathelicidins from neutrophils is expected, as these leukocytes infiltrate the lamina propria during colitis^27,76–80^ and produce higher amounts of the peptide stored in secondary granules.^23,27,81^ Cathelicidin production from epithelial cells likely played a minor role in the mucus barrier. However, the production of cathelicidin by colonic epithelium infected with *C. rodentium* could be crucial for direct antimicrobial defenses or other local signaling mechanisms, as the peptide is overexpressed in the epithelium during infections.^82^ Thus, cathelicidins may have compartmentalized functions, with immune cell-derived CRAMP activating ROS synthesis and influencing mucus secretion and other goblet cell proteins through distinct mechanisms compared to epithelial-derived CRAMP. It is noted that although *Camp*^*−/−*^ mice infected with *C. rodentium* showed some increased gut permeability, they did not manifest severe histological colitis. It is likely that upregulation of compensatory mechanisms, including a transcriptional activation of macrophages and upregulation of other antimicrobial peptides (i.e. *Rnase6*) in the colon, could have prevented exaggerated tissue damage. In agreement, macrophage activation through pathways such as EGFR signaling, autophagy, and M2 polarization enhanced *C. rodentium* clearance while mitigating tissue damage.^83^

The lack of endogenous cathelicidin impeded microbiome responses expected in intestinal inflammation and infection. The microbiota in C*amp*^*-/-*^ littermates did not change significantly during *C. rodentium* infection. However, during infection with *C. rodentium* in *Camp*^*+/+*^ littermates there was a shift in microbiota with reduced *Cyanobacteria* and *Verrucomicrobiota* and increased Shannon alpha diversity, consistent with previous *C. rodentium* studies.^84^ The abundant population of *Verrucomicrobiota*, including *Akkermansia muciniphila*, in *C. rodentium*-infected *Camp*^*-/-*^ littermates may exacerbate the intestinal barrier disruption, given its ability to degrade mucin^85^ and utilize it as a nutrient source.^86–88^ At the same time, a reduction in *Firmicutes* underscores a disruption in commensal microbes that leads to impaired butyrate production^89^ and predisposes *C. rodentium* infection. Remarkably, the relative stability of the microbiota in *Camp*^*−/−*^ mice following infection with *C. rodentium* contrasted with the pronounced shifts in *Camp*^*+/+*^ littermates, showing increases in *Actinobacteria*, *Firmicutes*, and *Verrucomicrobiota*. In agreement, studies showed that mice producing cathelicidin have a balanced microbiome, and exogenous cathelicidin enhances the colonization of beneficial bacterial taxa while repressing harmful ones.^90^ We speculate that, because CRAMP is an antimicrobial peptide, it might selectively influence specific microbial bacteria.^91^ In its absence, *Camp*^*−/−*^ mice may lack this selective pressure and display a relatively stable community. Alternatively, *Camp*-deficient mice may have developed microbial communities characterized by reduced modularity and increased co-oscillation between taxa, which are inherently resistant to pathogen-induced changes. Confirmation of these taxons, targeted colonization, and depletion experiments are required to determine the causal relationship between specific microbial taxa and mucus barrier integrity in CAMP deficiency.

In summary, this study proposes a new mechanism whereby cathelicidin regulates the secretion of mucus, TFF3 and RELMβ by goblet cells via internalization of intracellular NADPH oxidases and the production of ROS (Figure 10). Mice lacking cathelicidin will likely be distressed by intestinal mucosal barrier dysfunction, eventually leading to aberrant local inflammation and bacterial load. The deficiency in ROS, a central mediator in mucosal defenses, would exacerbate the vulnerability of mucosal protection, leading to increased bacterial colonization and inflammation. A persistence of cathelicidin in evoking the secretion of TFF3 and RELMβ from goblet cells might eventually deplete mucus stores and exacerbate colitis. For instance, excessive RELMβ via REGIIIβ reduces colonic *Lactobacilli* spp. and induces dysbiosis in *Muc2*^*-/-*^ mice.^69^ The role of cathelicidin in stimulating the release of goblet cell-associated proteins would occur in concert with other immunomodulatory properties of cathelicidin. For instance, the chemotaxis of neutrophils, T cells, and monocytes,^61^ the induction of phagocytosis in monocytes^92^ and the formation of neutrophil extracellular traps (NETs).^93,94^ Some of these cathelicidin functions would be specific in the gut, such as sensing Gram-negative bacteria/LPS and secretion of epithelial CXCL8 and local neutrophil recruitment^23^ and preventing epithelial apoptosis by sustaining Tollip,^29^ which explains the pleiotropic homeostatic functions of cathelicidin during DSS colitis,^41,95,96^ TNBS colitis,^97^ and *C. difficile* toxin A-inducing colitis.^98^ These immunoregulatory activities of cathelicidin may be physiologically more relevant than simple microbicidal effects, as they occur at sub-microbicidal concentrations ( < 2–4 μM),^99^ illustrating the complex biology of host defense peptides in gut biology.

## Supplementary Material

R1 Mirzadzare Table S2.xls

R1 Mirzadzare Table S1.xlsx

## Data Availability

The data supporting this study’s findings are available in open-access data repositories. Videos (Videos S1 and S2) are in Figshare at https://figshare.com/s/ad99aac22f11fd132b87. and https://figshare.com/s/86558a725d5ae2b5f764. The RNA seq raw data is in BioProject ID PRJNA967825 http://www.ncbi.nlm.nih.gov/bioproject/967825, and the primary transcripts and GO interactions are reported in Supplementary Tables S1 and S2.

## Highlights

- *Camp*^*-/-*^ littermates infected with *C. rodentium* retained mucin granules in bloated goblet cells.
- *Camp*^*-/-*^ littermates showed impaired mucus secretion and had decreased ROS biosynthesis.
- Goblet cells stimulated with LL-37 secreted TFF3 and RELMβ via a ROS-dependent mechanism.
- Cathelicidin regulated goblet cell mucus secretion and bacterial clearance.
